# Burden of Friedreich’s Ataxia to the Patients and Healthcare Systems in the United States and Canada

**DOI:** 10.3389/fphar.2013.00066

**Published:** 2013-05-22

**Authors:** Barbara Polek, M. J. Roach, William T. Andrews, Manfred Ehling, Sam Salek

**Affiliations:** ^1^Santhera Pharmaceuticals LtdLiestal, Switzerland; ^2^Santhera Pharmaceuticals IncCharlestown, MA, USA; ^3^Population, Microcensus, Housing and Migration/Federal Statistical Office of GermanyGermany; ^4^The Welsh School of Pharmacy, Cardiff UniversityCardiff, UK

**Keywords:** Friedreich ataxia, resource utilization, healthcare resource, rare diseases, cross-sectional studies, cost of illness

## Abstract

**Objective:** The study intended to substantiate healthcare resource utilization, costs, and funding patterns of US and Canadian Friedreich’s Ataxia (FRDA) populations, to assess compliance with treatment guidance and to identify areas where novel healthcare measures or improved access to existing care may improve patients’ functional and social capabilities and reduce the financial impact on the healthcare systems.

**Methods:** Healthcare resource utilization and costs were collected in a cross-sectional study in the US (*N* = 197) and Canada (*N* = 43) and analyzed across severity of disease categories. Descriptive statistics, correlation analysis, and hypothesis testing were applied.

**Results:** In the US, healthcare costs of FRDA patients were higher than those of “adults with two and more chronic conditions.” Significantly higher costs were incurred in advanced stages of the disease, with paid homecare being the main driver. This pattern was also observed in Canada. Compliance with the recommended annual neurological and cardiological follow-up was high, but was low for the recommended regular speech therapy. In the US public and private funding ratios were similar for the FRDA and the general populations. In Canada the private funding ratio for FRDA was higher than average.

**Conclusion:** The variety of healthcare measures addressing the broad range of symptoms of FRDA, and the increasing use of paid home care as disease progresses made total US healthcare costs of FRDA exceed the costs of US adults with two and more chronic conditions. Therefore, measures delaying disease progression will allow patients to maintain their independence longer and may reduce costs to the healthcare system. Novel measures to address dysarthria and to ensure access to them should be further investigated. The higher than average private funding ratio in Canada was due to the relatively high cost of the pharmacological treatment of FRDA.

## Background

### Rare disease

Friedreich’s Ataxia (FRDA) is one of the 6000–8000 rare diseases currently known. It occurs in Caucasians with an average prevalence of 3–4 in 100,000 cases (Schulz et al., 2009), a figure well below the threshold set by orphan drug regulations in Europe and the United States to classify a disease as rare.

### Clinical picture

Friedreich’s Ataxia is a rare, inherited, progressive neurodegenerative multi-system disease that is characterized by a debilitating loss of balance and coordination in all four limbs, which leads to dependence on a wheelchair usually within 10–15 years after disease onset. Cardiac involvement is seen in most patients, with cardiomyopathy occurring mostly in patients with early disease onset for whom it may be life threatening. Skeletal abnormalities such as scoliosis and pes cavus are common. About 10% of the patients develop diabetes mellitus. Dysarthria (slow, jerky speech with sudden utterances) is seen in almost all patients, usually beginning shortly after disease onset and progressing until speech becomes almost unintelligible. Vision loss is experienced frequently, as is hearing impairment. Hearing loss is mainly seen in later stages of the disease. Disease onset usually takes place in the first two decades of life, but later onsets are seen, too. The further progress is unremitting, but frequently includes periods of stability at the beginning of the illness. Life expectancy of persons with FRDA is reduced to 40–50 years (Pandolfo, 2009; Schulz et al., 2009).

### Diagnosis and treatment

Diagnosis is made based on clinical symptom presentation and with confirmation of FRDA by genetic testing. Treatment is largely symptomatic. Supportive care is provided by a wide variety of healthcare practitioners to help with Activities of Daily Living and to increase the health related quality of life of the affected persons. In addition, patients in Canada have had access to the orphan drug Catena^®^ (INN: idebenone), the first pharmacotherapy for FRDA, since its approval in 2008. (This approval was withdrawn from the Canadian market by the end of April 2013 due to the failure of additional clinical studies to confirm the efficacy claim.) At the time of the study, Catena^®^ was not approved in the United States. Neurologists, cardiologists, general practitioners, ophthalmologists, and other specialists are consulted for diagnosis and ongoing management of the neurological and non-neurological symptoms of FRDA. Physiotherapy, speech therapy, and other non-physician services address functional impairments. The range of medical devices used includes wheelchairs and walking aids, braces for the feet and spine, and hearing aids. Surgery may be required to address skeletal deformities, and cardiac conditions may lead to hospitalizations. Adaptations to the car and home are made to facilitate the patients’ moving about. Pharmacological agents are taken to manage conditions of the nervous, musculoskeletal, cardiovascular, and the genitourinary systems and of the metabolism, to name the most important areas. And finally, as the disease progresses and family and friends may no longer be able to cope with the challenges of the care, paid homecare and stays in long-term care facilities may become necessary (Schulz et al., 2009).

## Objectives

The present study intended to enhance the healthcare community’s understanding of FRDA patients’ healthcare resource utilization and related cost impact on the concerned healthcare systems. The rate of monitoring visits to neurologists and cardiologists, as well as the extent of utilization of physio and speech therapy addressing ataxia and dysarthria, most characteristic and highly disabling symptoms of FRDA, were assessed for compliance with treatment guidance established by a panel of European experts (Schulz et al., 2009). The data were also evaluated with the goal to identify areas where novel healthcare measures or improved access to existing care may enhance patients’ functional and social capabilities and reduce the financial impact on the healthcare systems. The private and public funding ratios were compared for persons with FRDA and the general population to identify any discrepancies. This study came second to a study on healthcare resource utilization of FRDA patients in the United Kingdom and Germany (Giunti et al., 2013).

## Methods

### Study design and measured concepts

Retrospective, cross-sectional surveys were conducted in the United States and Canada in persons with FRDA. The observation period covered 12 months, most of which was in 2010. The study adopted the perspective of the healthcare systems. Therefore, measured concepts were limited to direct medical and non-medical costs which were paid by public and private insurance, as well as out of pocket by the patients and their families. They included a total of 11 healthcare components: physician and therapist services, laboratory analyses, prescription medicines, devices, hospital admissions including the use of emergency rooms, car and home adaptations, paid homecare, and stays in long-term care facilities.

### Survey samples

Estimates based on published prevalence ratios suggest the population of FRDA to amount to between 5000 and 10,000 persons in the United States and between 300 and 750 persons in Canada. To mitigate anticipated difficulties to achieve adequate sample sizes when using a limited number of insurance claims databases the survey participants were recruited from patient registries: in the United States, the patient registry of FARA, the Friedreich Ataxia Research Alliance, a large not for profit organization dedicated to furthering research in FRDA, was used. The Canadian survey recruited the participants among the registrants of a patient support program offered to patients who sought treatment with Catena^®^. Inclusion criteria in both surveys consisted of a genetic confirmation of FRDA and residence in the respective country. Adult caregivers were allowed to fill the questionnaire on behalf of minor patients.

### Questionnaire

To enhance the content validity of the questionnaire it was built to reflect treatment recommendations set up by a panel of experts from a European perspective (Schulz et al., 2009). The lack of treatment guidelines specifically taking a US and/or Canadian perspective was compensated for by having the survey questionnaire reviewed and approved by the Executive Director of the Friedreich Ataxia Research Foundation FARA, located in the US, and by an expert genetic counselor in Canada. Twenty one closed questions addressed the diagnostic, medical treatment, and care options which would most widely be used by persons with FRDA. To further construct validity the respondents were offered predefined frequency options to inform about the level of use they made of the different services during the observation period. The questionnaires were tested by patients and medical experts.

### Study procedures

The patients were asked to fill either an electronic or a paper questionnaire to inform about selected demographics, their current status of disease severity (measured as degree of wheelchair use), as well as their quantitative utilization of resources across 11 healthcare components. With the exception of the costs of car and home adaptations, patients were not asked to report costs. For the US survey these were sourced from the Centers of Medicare & Medicaid Services CMS, Red Book, published literature (Prudential Research Report, 2010; Univita Health, 2010) and internet vendor price lists. For the Canadian survey, the resource costs were sourced from provincial benefit schemes and drug formularies (Ontario Ministry of Health and Long-term Care, 1999; Régie d’Assurance Maladie de Québec (RAMQ), 2008a,b; Ontario Ministry of Health and Long-term Care, 2010a; Ontario Ministry of Health and Long-term Care, 2010b; Ontario Ministry of Health and Long-term Care, 2010c; Ontario Ministry of Health and Long-term Care, 2010d), the Canadian Institute for Health Information (2010a,c), professional associations and internet vendor price lists. Costs which dated earlier than 2010 were inflated by the appropriate price index. Patient reported expenditures for car and home adaptations in excess of 1000 US or Canadian dollars were converted to equivalent annual costs with a useful life of *n*_c_ = 4 years for car adaptations and *n*_h_ = 15 for home adaptations and an interest rate of *r* = 7%. The quantities of measures of care reported by the patients were multiplied by these cost rates to determine total costs by type of service, by component and on the summary level of total healthcare costs. To estimate the ratios of costs borne by public and private payers, the costs of each healthcare component were multiplied by public and private funding ratios specific to each component, as well as by the public and private funding ratios observed for the total healthcare costs of the general population. Public and private funding ratios for the US were sourced from the Medical Expenditure Panel Survey MEPS (U.S. Department of Health and Human Services, 2008a,b) and published literature (Tumlinson et al., 2007), and for Canada, from Canadian Institute for Health Information (2005) and Health Canada (2001). The studies were approved by Western Institutional Review Board (WIRB).

## Analyses

### Healthcare costs

Descriptive statistics were used to characterize the distribution of costs by healthcare component and on the summary level, both for the entire patient population and by severity of disease category. The significance of cost differences between severity of disease categories was assessed by means of the Mann Whitney test. To put FRDA related healthcare costs into perspective for the US, they were compared with the healthcare costs measured for the comparator population of “US adults with two and more chronic conditions” (Machlin et al., 2008). Correlation analysis was used to test the association of severity of disease with total healthcare costs.

### Compliance with treatment recommendations

Schulz et al. (2009) recommended patients to see a neurologist and a cardiologist at least for one annual neurological, cardiological, and hematological evaluation, and an ongoing physiotherapy and regular speech therapy to address ataxia and dysarthria, the most characteristic symptoms of FRDA. Compliance was considered high if 70% or a higher percentage of respondents reported at least one visit per year to a neurologist and/or a cardiologist, as well as the performance of one laboratory panel analysis. Compliance of the reported frequency of therapist visits was assessed to be high if a majority of the patients reported an at least monthly frequency of visits.

### Payer analysis

To determine if public and private funding ratios for the healthcare costs incurred by persons with FRDA were different from the public and private funding ratios observed for the general population, the Wilcoxon signed rank test was applied.

The statistical analysis was performed with IBM^®^ SPSS^®^ Statistics version 19.0.

## Results

### Demographics, response rate

#### United States

Of the 789 persons in the US who received a link to the electronic questionnaire 197 submitted a valid data record, which implies a response rate of 25%. The mean age of the respondents was 30 years (28–32 years, 95% CI) with the age range spanning 4–72 years. About 92% of the respondents were younger than 55 years, which reflected the average life expectancy of 40–50 years reported in the literature for this patient population (Pandolfo, 2009; Schulz et al., 2009). The median age of the survey participants (26 years) was considerably lower than the median age of the US resident population (36.8 years). Disease onset was reported by 71% of the respondents to have occurred at an age of 20 years and earlier, confirming the observations reported by Pandolfo (2009). The respondents’ locations were spread across 21 of 50 US states, and 52.2% of the respondents were living in the 10 states which in 2010 hosted 50.2% of the US population (U.S. Census Bureau, 2010).

#### Canada

Of the 87 Canadian FRDA patients who explicitly accepted to receive a questionnaire, 57 (65.5%) returned a valid data record. However, only 43 (49.4%) returned the questionnaire before the database was closed and so were considered for the analysis. The mean age of the respondents was 27 years (23–31 years, 95% CI), and the age range spanned 6–64 years. All but one respondent were 50 years old or younger. Disease onset for 74.4% of the respondents happened at an age of 20 years and earlier. The respondents resided in the seven Canadian provinces and territories which accounted for about 95% of Canada’s population in 2006. A majority of 27 persons or 63% of all respondents lived in Québec.

The demographics observed in the US and Canadian samples suggested that these were fairly representative of the FRDA populations.

### Severity of disease

The respondents classified their current status of disease severity on a scale including four levels of ambulatory capability ranging from “no wheelchair use” to “use of a wheelchair all the time.” For the further analysis the four groups were collapsed into two categories of “less affected persons” who used a wheelchair never or only occasionally, and of “severely affected persons” who used a wheelchair most or all the time. Eta values of 0.671 for the US and 0.864 for the Canadian sample identified a strong correlation between these severity of disease categories and disease duration (years since diagnosis). The logistic regression analysis conducted on the US sample confirmed this observation. The test included disease duration, age, regular use of idebenone (Catena^®^ and idebenone products from uncontrolled sources), as well as the interactions between these potential predictor variables and showed that disease duration correctly predicted the severity of disease category in about 80% of the cases, whereas the other variables exhibited no predictive power. This finding was accepted as a corroboration of the assumption that the increasing degree of wheelchair use over time reflects the progressive nature of this disease.

### Healthcare costs

Eta values of 0.290 in the US and 0.309 in the Canadian sample indicated a low moderate correlation of severity of disease and total healthcare costs.

#### United States

**Figure 1:**
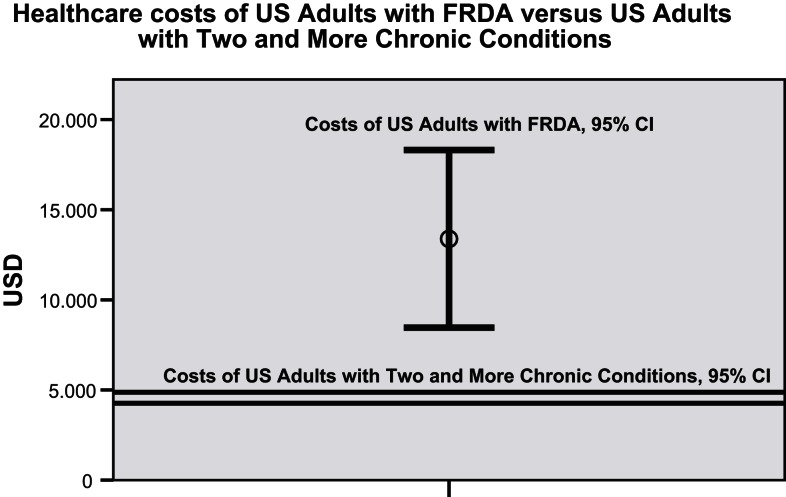
**Comparison of mean healthcare costs of US adults with FRDA and of US adults with two and more chronic conditions (95% CI)**. (N.B.: the analysis of healthcare costs of US adults with two and more chronic conditions by Machlin et al. (2008) was based on MEPS data which included more than 30,000 individuals. The considerably different sample size of the compared populations contributed to the different sizes of the 95% confidence intervals).

**Figure 2:**
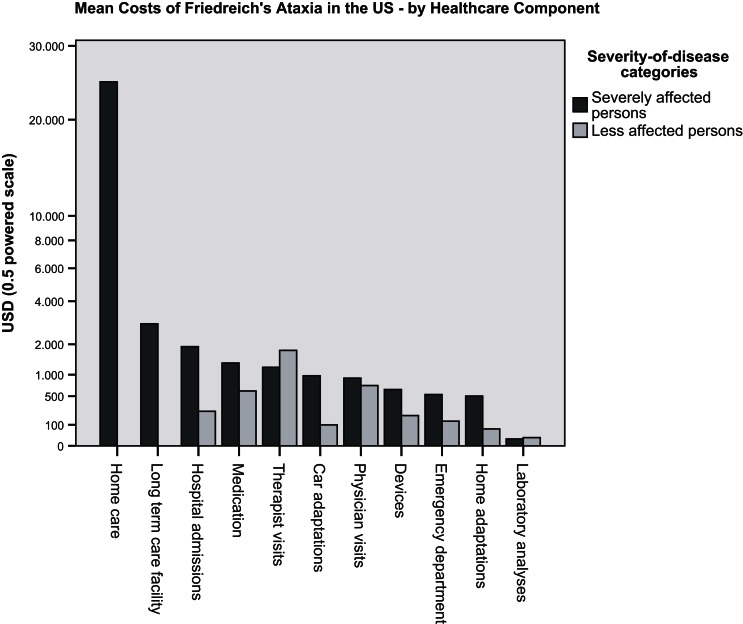
**US survey: mean costs of persons with FRDA, by healthcare component and severity of disease category**.

The frequency distribution of total healthcare costs in the US showed a distinct positive skew with the mean costs of 12,850 USD (9224 to 16,477 USD; 95% CI) about three times higher than the median costs. To generate a common basis for the comparison with mean healthcare costs established for US adults with two and more chronic conditions (Machlin et al., 2008), data records of FRDA persons of 18 years and younger had to be excluded from the total sample, and the costs of devices, car and home adaptations and of stays in long-term care facilities had to be disregarded. Figure 1 illustrates that FRDA related costs (8458 to 18,307 USD; 95% CI) were distinctly higher than the costs measured for the comparator population (4266 to 4876 USD; 95% CI). The difference was still clear when the 2005 mean costs of the comparator population (4571 USD) were inflated to 2010 (6217 USD) by applying the Medical Care component of the US Consumer Price Index (inflated costs not shown in Figure 1). Paid homecare accounted for the major portion of costs of patients with annual healthcare costs of more than 100,000 USD. All highest cost cases belonged to the category of severely affected persons. Figure 2 presents the mean costs by healthcare component for each of the severity of disease categories. The graph confirms the dominant role the costs of paid homecare and of stays in long-term care facilities play for severely affected persons, whereas the less affected persons incurred highest mean costs for therapist visits, physician visits, and medication. To test the significance of cost differences between severity of disease categories, Mann Whitney tests were applied. Costs were significantly higher for the severely affected than for the less affected persons for homecare (*p* < 0.01), prescription medicines (*p* < 0.05), and car adaptations (*p* < 0.01). The costs of therapist visits were significantly higher for the less affected persons (*p* < 0.05). The cost differences for all other components were not found to be statistically significant.

The dispersion of total healthcare costs was almost four times larger for the severely affected persons (cost range of 0–181,131 USD) than for the less affected persons (cost range of 100–47,541 USD).

#### Canada

**Figure 3:**
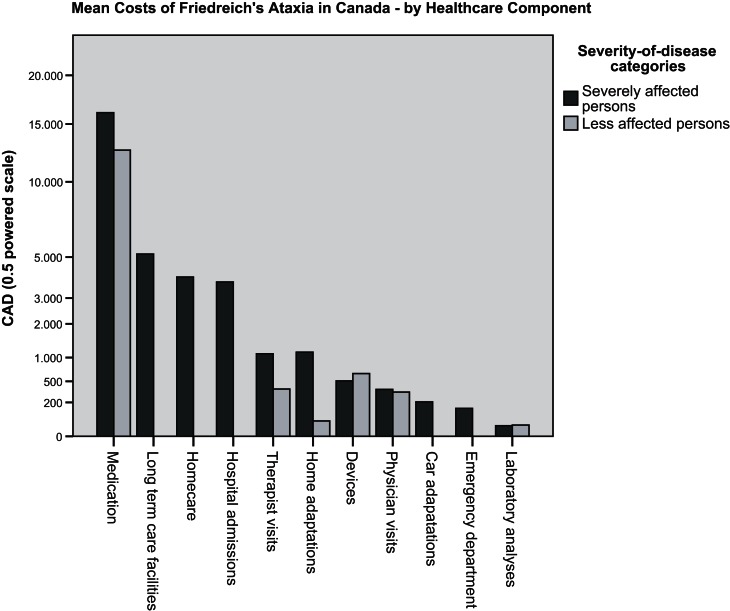
**Canadian survey: mean costs of persons with FRDA, by healthcare component and severity of disease category**.

The frequency distribution of total healthcare costs measured in persons with FRDA living in Canada was symmetric, as opposed to the positively skewed frequency distribution observed in the US. The mean costs of 34 683 CAD (26 698–42 668 CAD; 95% CI) were about three times higher than the mean costs measured in the US survey. This was mainly due to Catena^®^ with annual costs of about 38 000 CAD. (At the time of this survey, Catena^®^ was not approved in the United States). The costs of medication, and particularly of Catena^®^ which was regularly taken by members of both severity of disease categories, was the main reason for the shift of the median cost (38 826 CAD) to a value close to and even higher than the mean costs. Medication costs were the main driver of the highest costs reported for less affected persons, and contributed to the highest costs observed in severely affected persons, together with the costs for homecare and long-term care facilities. Figure 3 presents the mean costs by healthcare component for each of the severity of disease categories. It confirms the dominant role of medication costs in both severity of disease categories. When medication costs were disregarded, the pattern of costs by component was similar to the one observed in the US: costs of the severely affected persons were driven by paid homecare and institutional care, whereas the costs of the less affected persons were driven by devices, therapist and physician services. The results of the Mann Whitney tests revealed that the significant difference of the distributions of total healthcare cost across the two severity of disease categories (*p* < 0.05) was entirely due to homecare costs which were found to be significantly higher in severely affected persons than in less affected persons (*p* < 0.01). As was observed in the US survey, the dispersion of total healthcare costs was larger for the severely affected persons (cost range of 329 to 99 752 CAD) than for the less affected persons (cost range of 258 to 49 270 CAD) albeit by a factor of about two compared to a factor of four measured for the US.

### Compliance with basic treatment recommendations

In the US, 86 and 77% of the respondents, respectively, saw a neurologist or a cardiologist, and 66% saw both of these specialists at least once per year. In Canada, the percentages were 75, 86, and 65% (Table 1). Somewhat lower figures of 72% of the respondents in the US and of 62% in the Canadian sample reported to have had a laboratory panel analysis done. However, in both countries, the response rate to this question was only about 30%. Therefore, the results might not be representative of the FRDA population. In the US, 51% of the respondents saw a physiotherapist at least one to two times a year, and 26% of the respondents saw their physiotherapist at least once a month. A clear majority of 83% of the respondents never saw a speech therapist, and only 3.5% of the respondents made regular monthly or more frequent use of a speech therapy. This was reflected in a five times lower average number of visits to speech therapists than to physiotherapists (Table 2). A similar pattern was observed in Canada where 59% of the respondents saw a physiotherapist at least one to two times a year and 24% saw the physiotherapist at least monthly. Eighty seven percent of the respondents never saw a speech therapist and none used this type of therapy on a regular basis.

**Table 1:** **Percentage of survey respondents who saw specialists and had a lab analysis done at least once during the 12-months observation period**.

	United States (%)	Canada (%)
Neurologist	86	75
Cardiologist	77	86
Neurologist + cardiologist	66	65
Panel of lab analyses	72	62

**Table 2:** **Mean number of visits of the survey respondents to different types of therapists during the 12-months observation period**.

	United States	Canada
Physiotherapist	10	6
Chiropractor	2	3
Speech therapist	2	0
Dietician	1	0

### Payer analysis

Public and private funding ratios for the general populations were almost inverse in the US (28%/72%) compared to Canada (70%/30%) (Table 3).

**Table 3:** **Public and private funding ratios in the United States and Canada for the general population and for persons with FRDA**.

	United States		Canada	
	Public (%)	Private (%)	Public (%)	Private (%)
General population	28	72	70	30
Persons with FRDA	34	66	65	35

In the US, the public share of FRDA costs was higher than the public share of the costs of the general population. However, the difference was not statistically significant. Homecare costs accounted for the most important portion of the public payer share. According to MEPS, in 2008, 86.5% of the costs of “all home services” were funded from public sources (U.S. Department of Health and Human Services, 2008a,b).

In Canada, the private share was significantly higher for FRDA’s healthcare costs than for the general population’s healthcare costs (35 versus 30%, *p* < 0.01). The driving force here was medication costs, which accounted for the most important portion of the privately funded healthcare costs. On average, 55% of prescription medicines costs were privately funded (Canadian Institute for Health Information, 2010b).

### Limitations of the study

Due to the lack of specific North American treatment guidance a European expert panel’s guidance was used to set up the survey questionnaire. The small number of medical experts in such a rare disease promotes a close research collaboration across continents. Whereas this allows to assume that the European treatment recommendations are also accepted in the US and Canada the country specific funding schemes influence the degree of access to the recommended healthcare services. Any interpretation of resource utilization across countries must therefore be accompanied by a notion of caution.

With the Canadian respondents sampled from the registry of patients participating in the Catena^®^ access program a selection bias was introduced which resulted from the fact that all of these patients had got a prescription for Catena^®^ and 53% of the participants reported regular use of Catena^®^, a pharmacological treatment not available to Canadian patients outside this sample frame or who did not have adequate private insurance nor qualified for the statutory health insurance. The healthcare cost estimates for the Canadian sample are therefore likely to be overstated.

Although measures were taken to support the respondents’ memory of healthcare services used during the 12-months observation period, the accuracy of recollection from such a long period of time may not have been as good as if a prospective patient diary had been used.

## Discussion and Conclusion

The results of the surveys substantiated an impressive breadth and level of care addressing the broad range of clinical symptoms associated with FRDA. It did not therefore come as a surprise that US residents with FRDA were observed to incur considerably higher mean healthcare costs than the comparator population of “US adults with two and more chronic conditions.” Nor was the marked cost impact which the availability of an orphan drug treatment for FRDA had in Canada an unexpected finding. The use of paid homecare and stays in long-term care facilities was observed to be a hallmark and a major cost driver of severe stages of the disease.

In the US the public versus private funding pattern for healthcare measures utilized by FRDA patients followed the pattern observed for the general population. In Canada where more than half of the prescription medicines’ costs are privately funded, the relatively high costs of Catena^®^, an orphan drug for the treatment of FRDA, made the private funding ratio for FRDA patients significantly exceed the private funding ratio observed in the general population. Limited availability of private funding may have been the reason that only about half of the patients with a prescription for this drug were actually taking it regularly.

Novel healthcare measures resulting in a slowed progression of the disease may help to preserve patients’ independence longer and bear the potential of reducing the costs to the healthcare systems. In addition, a more regular management of dysarthria may contribute to enhancing the patients’ social capabilities. Reasons for the relatively low utilization of speech therapy should be further investigated with the goal to remove obstacles which may impair the accessibility of speech therapy and to identify further clinical measures to address this characteristic symptom of FRDA.

## Conflict of Interest Statement

The authors declare that the research was conducted in the absence of any commercial or financial relationships that could be construed as a potential conflict of interest.

## Supplementary Material

The Supplementary Material for this article can be found online at: http://www.frontiersin.org/Pharmaceutical_Medicine_and_Outcomes_Research/10.3389/fphar.2013.00066/abstract
