# Supplementary material for: Burden of Friedreich’s Ataxia to the Patients and Healthcare Systems in the United States and Canada
Source: Front Pharmacol. 2013 May 22;4:66. doi: 10.3389/fphar.2013.00066 (PMC3660667; doi:10.3389/fphar.2013.00066)
Supplement: Supplementary file 1 [file 48722_Salek_DataSheet1.PDF]

US Questionnaire

Note: The following questionnaire text served as script for the setup of the questionnaire on the web. The first screen contained the Informed Consent or “Research Subject Information Sheet”, as it was called on the ethics committee’s (Western IRB WIRB) template. Most answer selections were programmed as drop-down lists.

Press the “AA” button on the upper right edge of the screen to increase the font size.
Press the button once to change from small to medium font size, and a second time to change from medium to large font size.

Research Subject Information Sheet

**Sponsor:**	Santhera Pharmaceuticals (USA) Inc
**Protocol title:**	Healthcare Resource Utilization of Persons with Friedreich’s Ataxia living in the United States
**Investigator:**	Barbara Polek, Santhera Pharmaceuticals (Switzerland) Ltd

In this information sheet and the accompanying questionnaire, “you” always refers to the person who has Friedreich’s Ataxia.

You are being asked to be in a research study that will try to advance the understanding of the type, quantity and approximate costs of the medical care used on average by persons with Friedreich’s Ataxia.

Your participation will involve your filling a questionnaire that asks you to inform about medical products and services that you have been using during the last 12 months to manage your Friedreich’s Ataxia. Any medical resources used to assess or treat conditions other than Friedreich’s Ataxia should not be included in your answers.

If you are less than 18 years old, your parents or other caregiver may fill the questionnaire on your behalf.

It will take you about 20 minutes to complete the questionnaire.
It is planned to include about 250 subjects in this research.

There are no known risks associated with being in this research, and you will not receive a direct benefit if you agree to participate. However, the sponsor will make a donation of $25 to FARA for every completely filled questionnaire.

Your participation in this research is voluntary. Your refusal to participate or your decision to stop filling the questionnaire at any time will involve no penalty or loss of benefits to which you might otherwise be entitled. Your alternative is to not participate in this study.

Contact FARA at info@curefa.org or 484-875-3015 for questions about the research or if you think you have been harmed as a result of joining this research. Contact the investigator at barbara.polek@santhera.com for questions about the research. Contact Western Institutional Review Board (WIRB) if you have questions about your rights as a research subject: 800-562-4789. WIRB is a group of people who perform independent review of research.

The information about you that will be collected for this research will be kept anonymous to all parties involved in the research: FARA will not see the answers that you provide in the questionnaire. The sponsor, the investigator and any organization the sponsor may involve to compute and analyze the data will not know your identity. Your set of answers will be identified to the sponsor, the investigator and his contractors only by means of the individualized URL link to the web-based questionnaire.

FARA will have unrestricted access to the aggregated results of the survey.

By submitting a completed or a partly filled questionnaire, you accept that the sponsor or any organization contracted by the sponsor may use the data for the planned research. You will not have the possibility to recall any answers once you have submitted them.

If you decide to participate in the research and to fill the questionnaire, please confirm that you have read and understood this information by ticking the box below. Then press the SUBMIT button to open the questionnaire.
If you decide not to participate in the research, you may exit the application now.

☐ I have read and understood the information.

SUBMIT

Part A	Demographics
	**Please complete the questionnaire to the best of your ability. Try to answer all questions. If you don't know the answer to a question, you may skip the question.**<br>**Please note that once you have pressed the SUBMIT button you cannot go back to the question nor to any other previous question.**<br><br>**If you are a parent or caregiver and fill the questionnaire on behalf of a person with Friedreich's Ataxia, please note that all answers should refer to the person with Friedreich's Ataxia, and not to yourself.**
**1**	In which state do you live?<br>• Alabama<br>• Alaska<br>• …<br>• …
**2**	How old are you? __________ years
**3**	What is your gender?<br>Female ☐<br>Male ☐
**4**	What is your weight?<br>__________ pounds
**5**	What is your height?<br>__________ feet __________ inches

**6**	Please tick the description which best describes your current situation in regards to wheelchair use:<br><br>I do not use a wheelchair…………………………………………… ☐<br><br>I use a wheelchair only occasionally ………………… ☐<br><br>I use a wheelchair most of the time ………………… ☐<br><br>I use a wheelchair all the time ……………………… ☐
**7**	How old were you when a physician first diagnosed your Friedreich's Ataxia?<br>(The question refers to the date of the first diagnosis rather than the first symptoms.)<br><br>__________ years
**8**	Who is the doctor that you consult for the management of your Friedreich's Ataxia?<br><br>General practitioner ☐<br><br>Neurologist ☐<br><br>Paediatrician ☐<br><br>Other ☐

Part B	Visits at physicians and therapists
	**Your answers to the following questions should refer to physician visits related to your Friedreich's Ataxia only. They should not include visits that were unrelated to your Friedreich's Ataxia.**
**9**	**During the last 12 months:**<br>How often have you seen a **General Practitioner**?<br><br>Please select<br>• <blank> *(= default)*<br>• Never<br>• Once or twice<br>• 4 to 6 times<br>• About once a month<br>• About once a week
**10**	**During the last 12 months:**<br>How often have you seen a **Paediatrician**?<br>(A pediatrician treats children and young adults up to about 18 years.)<br><br>Please select<br>• <blank> *(= default)*<br>• Never<br>• Once or twice<br>• 4 to 6 times<br>• About once a month<br>• About once a week
**11**	**During the last 12 months:**<br>How often have you seen a **Neurologist**?<br>(A neurologist would – among other items - examine your abilities to stand, sit and walk)<br><br>Please select<br>• <blank> *(= default)*<br>• Never<br>• Once or twice<br>• 4 to 6 times<br>• About once a month<br>• About once a week

**12**	**During the last 12 months:**<br>How often have you seen an **Orthopaedist**?<br>(An orthopaedist is a surgery specialist concerned with the skeletal system or bones.)<br><br>Please select<br>• <blank> *(= default)*<br>• Never<br>• Once or twice<br>• 4 to 6 times<br>• About once a month<br>• About once a week
	**Your answers to the following questions should refer to therapist visits related to your Friedreich's Ataxia only. They should not include visits that were unrelated to your Friedreich's Ataxia.**
**13**	**During the last 12 months**:<br>How often have you seen your **therapists**?<br><br>Please tick the appropriate box for each type of therapist.

	Never	Once or twice	4 to 6 times	About once a month	About once a week
**Physical therapist**	☐	☐	☐	☐	☐
**Chiropractor**	☐	☐	☐	☐	☐
**Speech therapist**	☐	☐	☐	☐	☐
**Dietician**	☐	☐	☐	☐	☐
**Others**	☐	☐	☐	☐	☐

	**Your answers to the following questions should refer to physician visits related to your Friedreich's Ataxia only. They should not include visits that were unrelated to your Friedreich's Ataxia.**
**14**	**During the last 12 months:**<br>How often have you seen an **Ophthalmologist**?<br>(An ophthalmologist is a specialist of the eyes.)<br><br>Please select<br>• <blank> *(= default)*<br>• Never<br>• Once or twice<br>• 4 to 6 times<br>• About once a month<br>• About once a week
**15**	**During the last 12 months**:<br>How often have you seen an **Urologist**?<br>(The urologist specializes on conditions affecting the kidneys, the bladder and the genital organs.)<br><br>Please select<br>• <blank> *(= default)*<br>• Never<br>• Once or twice<br>• 4 to 6 times<br>• About once a month<br>• About once a week
**16**	**During the last 12 months:**<br>How often have you seen a **Cardiologist**?<br>(A cardiologist is a specialist for the heart.)<br><br>Please select<br>• <blank> *(= default)*<br>• Never<br>• Once or twice<br>• 4 to 6 times<br>• About once a month<br>• About once a week

Part C Medical procedures

17

During the last 12 months:
Did you ever receive care in an **Emergency Department** in a hospital for reasons related to your Friedreich's Ataxia?

YES ☐

NO ☐

If your answer was NO: → **Press SUBMIT to go to next page**

If your answer was YES:

For each of your visits to an Emergency Department (**ED**), please mark the reason.

**Emergency Department visit number**	**The reason was related to my Friedreich's Ataxia**	**The reason was <u>not</u> related to my Friedreich's Ataxia**
Visit 1	☐	☐
Visit 2	☐	☐
Visit 3	☐	☐
Visit 4	☐	☐
Visit 5	☐	☐

18

During the last 12 months:
Were you ever admitted to a **hospital** for reasons related to your Friedreich's Ataxia?

YES ☐

NO ☐

If your answer was NO: → **Press SUBMIT to go to next page**
If your answer was YES:

For each of your hospital admissions, please select a reason.

<table>
<tr><th>Hospital admission number</th><td rowspan="11">(For each admission, a drop-down list will be shown with the options listed below.)

Please select
• <blank> (= default)
• Congestive Heart Failure
• Heart rhythm abnormality
• Injury due to a fall related to Friedreich's Ataxia
• Surgery of the back
• Surgery of a foot
• Other</td></tr>
<tr><td>Admission 1</td></tr>
<tr><td>Admission 2</td></tr>
<tr><td>Admission 3</td></tr>
<tr><td>Admission 4</td></tr>
<tr><td>Admission 5</td></tr>
<tr><td>Admission 6</td></tr>
<tr><td>Admission 7</td></tr>
<tr><td>Admission 8</td></tr>
<tr><td>Admission 9</td></tr>
<tr><td>Admission 10</td></tr>
</table>

19

During the last 12 months:
Have you had a **blood draw** related to the clinical care of your Friedreich's Ataxia?

YES ☐

NO ☐

**Part D Drugs and Devices**	
**20**	Please list the **medications** that you have taken **all the time during the last 12 months** to treat conditions related to your Friedreich's Ataxia.<br><br>If there are no medications that you have taken all the time, you may skip this question and press SUBMIT to go to the next page.

…

21

Please list the **medications** that you have taken **one or two times for a short period of time during the last 12 months** to treat conditions related to your Friedreich's Ataxia.
(Examples:
You may have taken antibiotics to treat a urinary tract infection that was caused by your Friedreich's Ataxia.
You may have taken pain killers when you got injured during a fall.)

If there are no medications that you have taken only one or two times for a short period, you may skip this question and press SUBMIT to go to the next page.

…

22

Please inform about your **current use of walking aids**.

**Type of walking aid**	
Canes or crutches	Please select<br>• <blank> *(= default)*<br>• I don't use<br>• I started use during the last 12 months<br>• I started earlier than 12 months ago<br>• I don't use but acquired such a device during the last 12 months
Walking frame	
Rollator (a walker with wheels)	
Manual wheelchair	
Automated wheelchair	
Power scooter for community transportation	

**23**	Please inform about your **current** use of any **braces for your feet or orthopedic shoes:**<br><br>Please select<br>• <blank> *(= default)*<br>• I don't use<br>• I started use during the last 12 months<br>• I started use earlier than 12 months ago<br>• I don't use but acquired such a device during the last 12 months
**24**	Please inform about your **current** use of any **braces on your back:**<br><br>Please select<br>• <blank> *(= default)*<br>• I don't use<br>• I started use during the last 12 months<br>• I started use earlier than 12 months ago<br>• I don't use but acquired such a device during the last 12 months
**25**	Please inform about your **current** use of **hearing aids**:<br><br>Please select<br>• <blank> *(= default)*<br>• I don't use<br>• I started use during the last 12 months<br>• I started use earlier than 12 months ago<br>• I don't use but acquired such a device during the last 12 months

**26**	**During the last 12 months**: Have you made **adaptations to your home** to help you move about?<br><br>YES ☐<br><br>NO ☐<br><br>If your answer was NO → **Press SUBMIT to go to next page**<br><br>If your answer was YES: Please provide the following details:<br><br>**Approximate** costs in US dollars: ________________<br><br>Amount paid by the patient or his/her family: ________________
**27**	**During the last 12 months**: Have you made **adaptations to your car** to help you move about?<br><br>YES ☐<br><br>NO ☐<br><br>If your answer was NO → **Press SUBMIT to go to next page**<br><br>If your answer was YES: Please provide the following details:<br><br>**Approximate** costs in US dollars: ________________<br><br>Amount paid by the patient or his/her family: ________________

**Part E**	**Home Care, Nursing Home / Long-Term Care Facility**
**28**	Do you use **paid personal care assistance in your home**?<br>(Such service is provided by individuals that come to your home and are paid for their service.)<br><br>YES ☐<br><br>NO ☐<br><br>If your answer was NO: → **Press SUBMIT to go to next page**<br><br>If your answer was YES:<br><br>**During the last 12 months**: Approximately, during how many months did you use this paid personal care assistance?<br><br>Please select the number of months:<br><br>• <blank> *(= default)*<br>• 1<br>• …<br>• 12<br><br>During the months when you used paid personal care assistance: How many hours per day did this individual come in on average?<br><br>Please select the average hours per day:<br><br>• <blank> *(= default)*<br>• 1<br>• …<br>• 24

**29**	Do you permanently live in a **nursing home or long-term care facility**?<br><br>YES ☐<br><br>NO ☐<br><br>If your answer was YES: When did you move to the nursing home or long-term care facility?<br><br>Please select the year:<br>• <blank> *(= default)*<br>• 2010<br>• 2009<br>• Before 2009<br><br>Please select the month:<br>• <blank> *(= default)*<br>• January<br>• …<br>• December
**END**	**This is the end of the questionnaire. Thank you very much for your help!**<br>**You may now exit the application.**
